# Engineered elicitin protein enhances *Phytophthora* resistance in plants by dual immune induction and pathogen inhibition

**DOI:** 10.1007/s44154-025-00272-3

**Published:** 2025-11-28

**Authors:** Zhengpeng Li, Yong Pei, Hao Zhou, Hui Wang, Yining Guo, Zhiyuan Yin, Daolong Dou

**Affiliations:** 1 Jiangsu Collaborative Innovation Center of Regional Modern Agriculture and Environmental Protection/Jiangsu Key Laboratory for Eco-Agricultural Biotechnology Around Hongze Lake, Huaiyin Normal University, Huai’an, 223300 China; 2 College of Plant Protection, Nanjing Agricultural University, Nanjing, 211800 China; 3 Academy for Advanced Interdisciplinary Studies, Nanjing Agricultural University, Nanjing, 211800 China

**Keywords:** *Phytophthora*, Sterol, Elcitin, Pattern-triggered immunity

## Abstract

*Phytophthora* pathogens are devastating agricultural threats that cannot synthesize sterols and must scavenge them from host plants. This study exploits their sterol auxotrophy by engineering a dual-function elicitin protein, SOJ5^V84F^, for enhanced disease control. The V84F mutation in the sterol-binding pocket of the *Phytophthora sojae* elicitin SOJ5 abolishes sterol binding but retains interaction with the pathogen's sterol-sensing receptor kinase SSRK1. SOJ5^V84F^ acts as a dominant-negative inhibitor: it competitively disrupts SSRK1-mediated sterol signaling (calcium influx, MAPK activation) and significantly inhibits *P. sojae* growth in an SSRK1-dependent manner. Crucially, SOJ5^V84F^ retains its ability as a microbe-associated molecular pattern to robustly elicit reactive oxygen species burst in soybean, pepper, tomato, and potato plants. Consequently, pre-treatment with SOJ5^V84F^ provided superior protection compared to wild-type SOJ5 against *P. sojae* in soybean, and against *Phytophthora capsici* and *Phytophthora infestans* in pepper, tomato, and potato under greenhouse conditions. This work demonstrates that engineered SOJ5^V84F^ combines direct pathogen inhibition with host immune activation, establishing a novel dual-mechanism strategy for protein-based biocontrol against sterol-auxotrophic oomycetes.

## Introduction

*Phytophthora* species are notorious oomycete pathogens responsible for severe diseases in a wide range of economically important crops worldwide (Kamoun et al. 2015). These pathogens cause devastating losses in plants, leading to significant agricultural and economic impacts. Potato late blight, caused by *P. infestans*, not only contributed to the 19th-century Irish Famine but continues to pose a significant threat to the secure production of potatoes and tomatoes to this day (Fry 2008; Nowicki et al. 2012). *P. sojae* is an economically important soybean-infecting pathogen that threatens global soybean production by causing root and stem rot diseases (Tyler 2007) while *P. capsici* has a broad host range and infects mainly vegetable crops (Quesada-Ocampo et al. 2023). Conventional chemical fungicides are often limited by environmental concerns, pathogen resistance, and regulatory restrictions, highlighting the urgent need for alternative, sustainable disease management strategies (Gisi and Sierotzki 2015; Quesada-Ocampo et al. 2023).

Unlike fungi, *Phytophthora* species are sterol auxotrophs and cannot synthesize sterols de novo (Gaulin et al. 2010; Wang et al. 2021). Instead, they rely on the acquisition of exogenous sterols from their host plants or the environment to support essential biological processes such as growth, reproduction, and pathogenicity (Wang et al. 2021; Pei et al. 2024). Our previous research has proven that *P. sojae* detects and utilizes host sterols through the membrane receptor complex SSRK1 (sterol-sensing receptor-like kinase 1) and elicitins (Pei et al. 2024, 2025). A key component of this sterol sensing system is the elicitin family of secreted proteins. Elicitins are ~ 10 kDa proteins conserved among *Phytophthora* and *Pythium* species (Derevnina et al. 2016). They were originally identified for their ability to elicit hypersensitive cell death in tobacco (Ricci et al. 1989). Elicitins are now recognized as microbe-associated molecular patterns (MAMPs) that trigger defense responses in a variety of plant species. ELR (elicitin response) from the wild potato species *Solanum microdontum* and REL (responsive to elicitins) from *Nicotiana benthamiana* have been identified as cell surface receptors mediating immune responses to elicitins (Du et al. 2015; Chen et al. 2023). Structurally, canonical elicitins contain a highly conserved 98-amino-acid domain with six cysteine residues forming three disulfide bridges. Also elicitins function as sterol carriers, transferring sterols from the host to the pathogen (Mikes et al. 1998; Boissy et al. 1999; Osman et al. 2001; Rodrigues et al. 2006). Importantly, multiple studies have shown that the sterol-binding activity of elicitins is dispensable for their ability to induce immune responses in plants. For example, elicitin mutants that fail to bind sterols still elicit cell death response, suggesting that sterol binding and defence elicitation are independent activities (Osman et al. 2001; Dokládal et al. 2012).

Our previous work demonstrated that the elicitin SOJ5 from *P. sojae* interacts with SSRK1 and mediates sterol-induced calcium signaling and MAPK activation in the pathogen. Interestingly, a point mutation (V84F) in the sterol-binding pocket of SOJ5 abolishes its ability to bind sterol but still interacted moderately with SSRK1 in the absence of *ß*-sitosterol. We hypothesized that this mutant elicitin might act as a dominant-negative inhibitor of SSRK1, thereby interfering with sterol sensing and impairing *Phytophthora* development. Here, we report that the SOJ5^V84F^ protein suppresses SSRK1-dependent sterol signaling and inhibits *P. sojae* growth. Despite lacking sterol-binding ability, SOJ5^V84F^ elicits robust immune responses in diverse plant species and provides enhanced protection against *Phytophthora* diseases in greenhouse assays. These findings establish a dual-function mechanism for elicitin-based disease control and identify SSRK1 as a promising target for protein-based biocontrol strategies.

## Results

### SOJ5^V84F^ protein SSRK1-dependently inhibits *Phytophthora sojae* growth

**Fig. 1:**
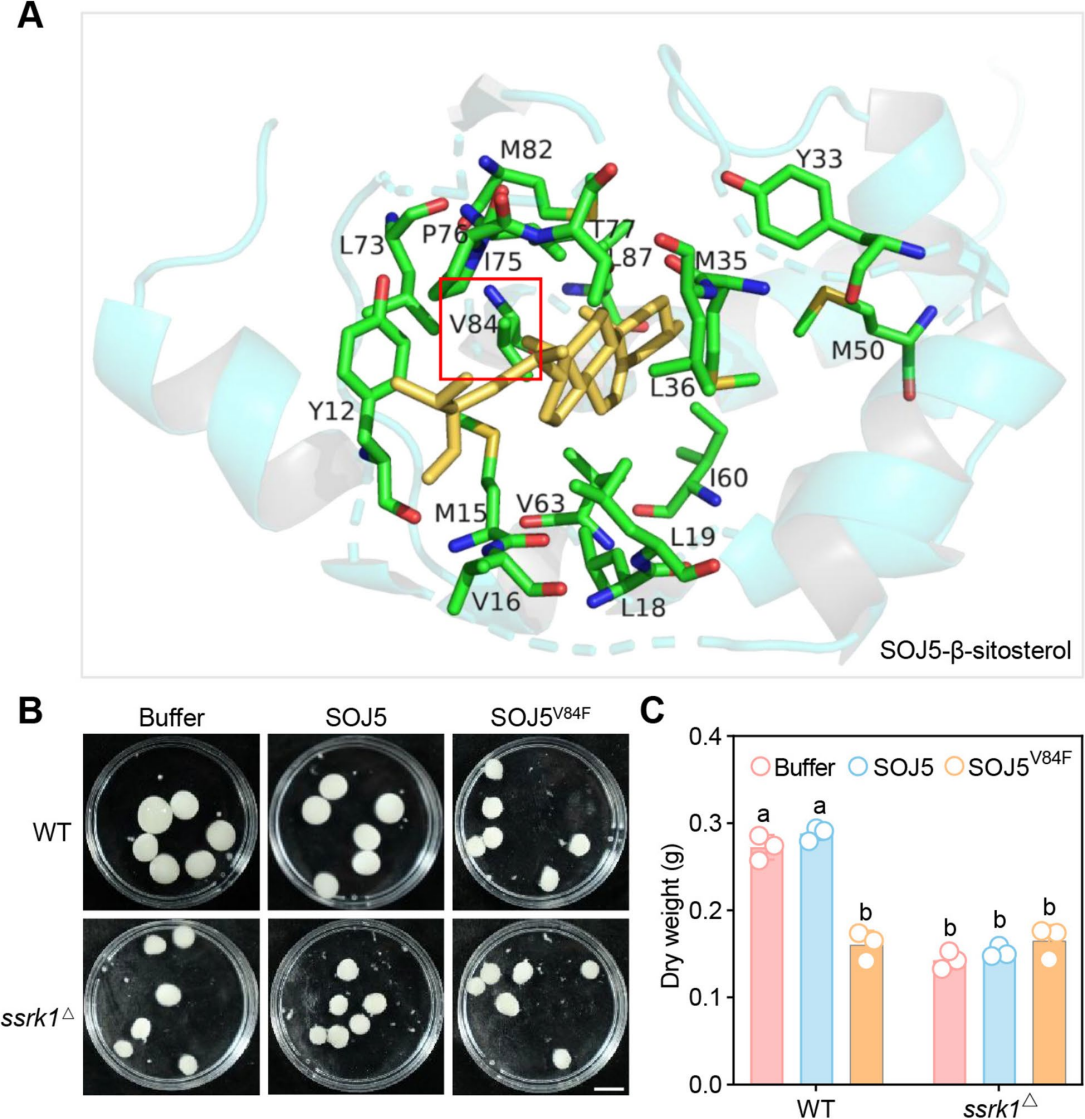
SOJ5^V84F^ suppresses the growth of *P. sojae*. **A** Structural models of the binding of SOJ5, SOJ1B and SOL7 to β-sitosterol predicted by molecular docking. **B** Six mycelial plugs of either *P. sojae* WT or *ssrk1*^△^ strains were incubated with SOJ5^V84F^ protein or buffer cultured on minimal medium supplemented with 25 μM *β*-sitosterol. The experiment was repeated three times with similar results. **C** Dry weight of *P. soaje* mycelial of different treatments. Different letters indicate values with statistically significant differences; two-way ANOVA

Previous structural studies have shown that elicitins contain a conserved hydrophobic pocket that accommodates sterol molecules (Boissy et al. 1999; Rodrigues et al. 2006). Val84 is positioned at the core of this pocket and stabilizes sterol insertion. Substitution of Val84 with a bulky phenylalanine side chain introduces steric hindrance, which disrupts proper sterol accommodation and abolishes sterol-binding activity (Dokládal et al. 2012). To illustrate this, we included a schematic representation of the elicitin–sterol complex highlighting the sterol-binding pocket and the position of Val84 (Fig. 1A). Considering that the sterol-non-binding mutant SOJ5^V84F^ still interacted moderately with SSRK1, at least in the absence of *ß*-sitosterol (Pei et al. 2024), we employed an excessive amount of SOJ5^V84F^ protein to induce a dominant-negative effect against other SSRK1-interacting elicitins. To assess the inhibitory effect of the sterol-non-binding mutant SOJ5^V84F^ on *P. sojae* growth, we examined mycelial development of *P. sojae* wild-type (WT) and SSRK1 knockout (*ssrk1*^∆^) strains in the presence of SOJ5^V84F^. As has been reported previously (Pei et al. 2024), the growth of *P. sojae* WT strain was strongly inhibited by 4 μM SOJ5^V84F^ protein (Fig. 1B-C). However, this inhibitory effect was largely comprised in the *ssrk1*^△^ strain, as revealed by the quantification of *P. sojae* biomass (Fig. 1B-C). These results indicate that the growth suppression by SOJ5^V84F^ requires functional SSRK1 and suggests a receptor-dependent inhibitory mechanism. Because all assays were performed in the presence of exogenous sterols, the observed inhibition reflects SSRK1-dependent interference with sterol signaling rather than sterol deprivation.

### SOJ5^V84F^ disrupts sterol-induced signaling in *Phytophthora sojae*

**Fig. 2:**
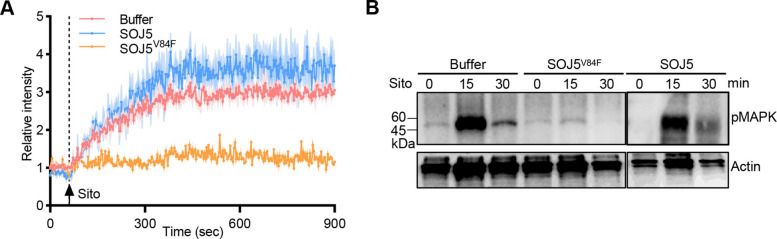
SOJ5^V84F^ suppresses sterol-induced responses in *P. sojae*. **A** Ca^2+^ dynamics were monitored using Fluo-4 AM in protoplast of indicated treatments following exposure to *β*-sitosterol (25 μM) after pre-treatment with SOJ5 or SOJ5^V84F^. **B** MAPK phosphorylation of *P. sojae* WT, following treatment with *β*-sitosterol, was evaluated after pre-treatment with SOJ5 or SOJ5^V84F^

To investigate whether SOJ5^V84F^ interferes with sterol-induced signaling in *P. sojae*, we measured calcium influx and MAPK activation in response to sterol treatment upon treatment with SOJ5^V84F^ in *P. sojae*. In normal conditions, sterol application triggered robust calcium influx and MAPK phosphorylation (Fig. 2A-B). However, pre-treatment with SOJ5^V84F^ significantly impaired both sterol-induced calcium influx (Fig. 2A) and MAPK activation (Fig. 2B). Although the V84F mutation reduces the affinity of SOJ5 for SSRK1, the mutant retains a sterol-independent basal interaction with the receptor. Unlike the wild-type protein, which delivers sterols to promote the formation of higher-order SSRK1–elicitin complexes and activate signaling, SOJ5^V84F^ binds SSRK1 without initiating complex assembly. Consequently, when supplied in excess, the mutant competes with sterol-loaded wild-type elicitins for receptor binding but prevents productive signaling, thereby exerting a dominant-negative effect. These findings reveal that SOJ5^V84F^ functions as a sterol signaling inhibitor targeting the pathogen directly.

### SOJ5^V84F^ enhances disease resistance in soybean

**Fig. 3:**
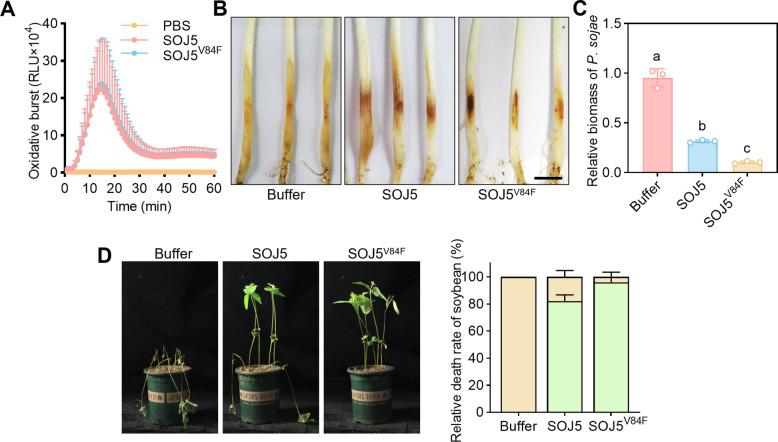
SOJ5^V84F^ enhances disease resistance in soybean. **A** The recombinant SOJ5 and SOJ5^V84F^ proteins induced similar oxidative burst in soybean. Leaf discs from soybean were assayed for reactive oxygen species production by measuring the relative light units (RLU) with a luminometer upon SOJ5 or SOJ5^V84F^ treatment for the indicated time points. **B** Etiolated soybean hypocotyls were soaked with 4 μM SOJ5 or SOJ5^V84F^ protein for 12 h. Lesions on soybean with different treatments at 48 h post inoculation (hpi) of 4-day-old etiolated hypocotyls with the indicated strains. **C** Relative biomass of *P. sojae* in the infected etiolated soybean hypocotyls was measured by qRT-PCR and was normalized to that treated with buffer (*n* = 3). Different letters indicate values with statistically significant differences; one-way ANOVA. **D** Effects of pre-treatment with SOJ5 or SOJ5^V84F^ protein on soybean disease caused by *P. sojae*

Previous studies showed that sterol-binding ability of elicitins is independent of inducing plant immune responses such as oxidative burst (Dokládal et al. 2012). We next assessed whether SOJ5^V84F^ still retains the ability to activate plant immunity, thereby conferring disease resistance. Both wild-type SOJ5 and the SOJ5^V84F^ mutant protein triggered comparable levels of reactive oxygen species (ROS) burst in soybean (Fig. 3A). Based on the discoveries that SOJ5^V84F^ can both induce resistance in soybean and exhibit antimicrobial activity by inhibiting sterol-induced responses, we further tested the roles in disease control. Pre-treatment with SOJ5 protein resulted in a significant reduction of *P. sojae* infection on soybean (Fig. 3B-C), because elicitins are also detected by plants to trigger plant immune responses. Interestingly, SOJ5^V84F^ exhibited significantly better efficacy in restricting *P. sojae* infection compared to SOJ5 proteins (Fig. 3B-C). Consistent with the infection assays with detached soybean hypocotyls, pre-treatment with SOJ5 or SOJ5^V84F^ both significantly reduced the numbers of dead soybeans caused by *P. sojae*, and SOJ5^V84F^ exhibited a better protection effect than SOJ5 (Fig. 3D).

### SOJ5^V84F^ treatment enhances resistance to other *Phytophthora* diseases in plants under greenhouse conditions

**Fig. 4:**
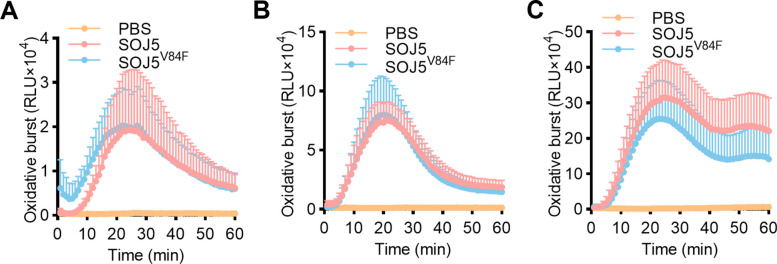
SOJ5 and SOJ5^V84F^ could induce comparable ROS burst in different plants. The recombinant SOJ5 and SOJ5^V84F^ proteins induced oxidative burst in pepper (**A**), tomato (**B**) and potato (**C**). Leaf discs from plants were assayed for reactive oxygen species production by measuring the relative light units (RLU) with a luminometer upon SOJ5 or SOJ5^V84F^ treatment for the indicated time points

In world, pepper and tomato are important vegetables suffering substantial losses from *P. capsici* infection (Nowicki et al. 2012; Quesada-Ocampo et al. 2023), and potato suffering more than 10 billion global yield losses due to the late blight pathogen *P. infestans* (Fry 2008; Nowicki et al. 2012)*.* Therefore, we also evaluated the efficacy of SOJ5^V84F^ in controlling disease caused by the two pathogens in three host plants*.* Firstly, we tested whether the ability of SOJ5 and SOJ5^V84F^ to activate plant immunity in above plants. The results showed that SOJ5 and SOJ5^V84F^ exhibit comparable ability to induce ROS production in pepper (Fig. 4A), tomato (Fig. 4B), and potato (Fig. 4C), indicating that the immune-activating function of elicitin is conserved across host species and maintained in the mutant protein.

**Fig. 5:**
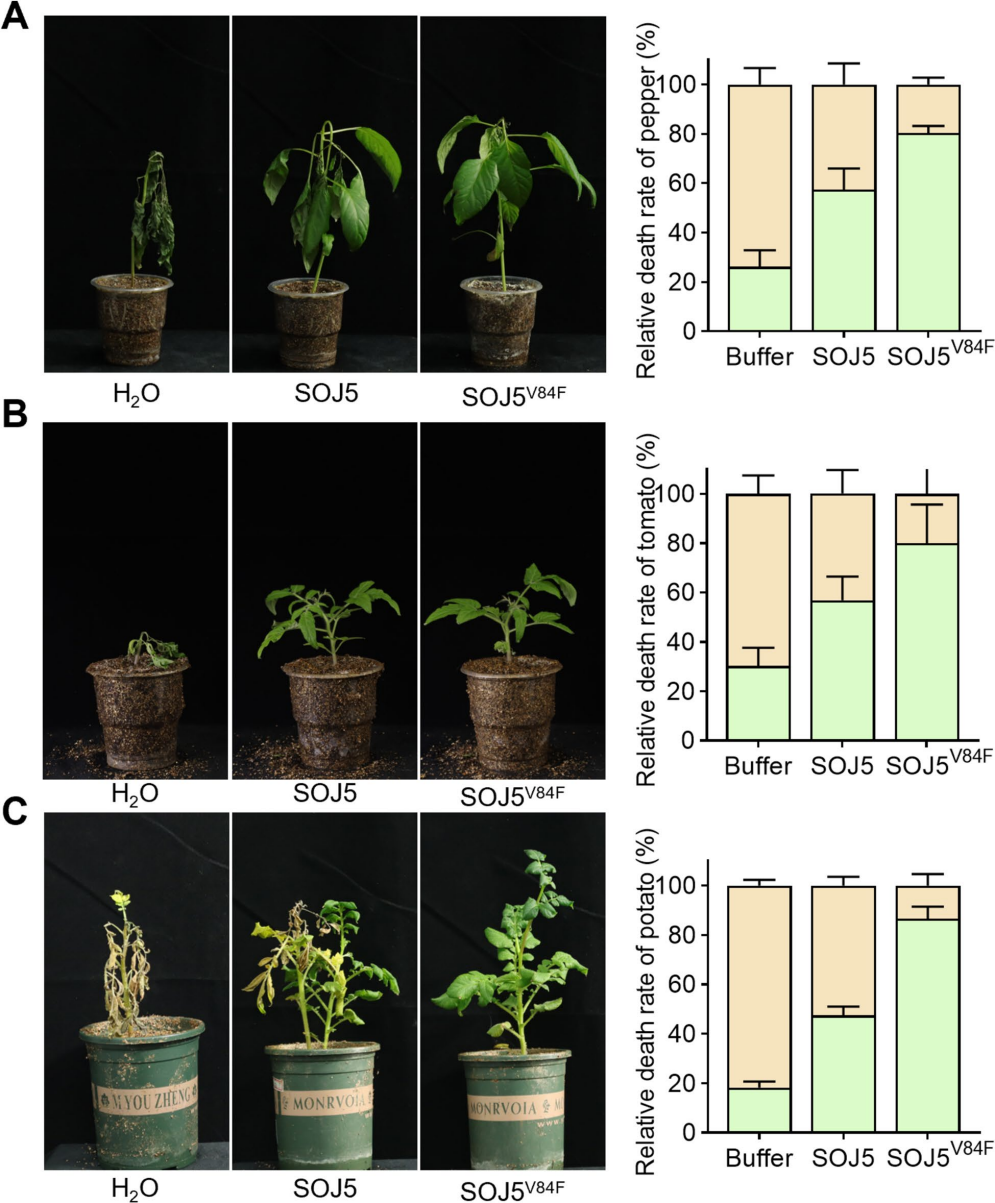
SOJ5^V84F^ enhances disease resistance in different plants. **A**-**C** Effects of pre-treatment with SOJ5 or SOJ5^V84F^ protein on (**A**) pepper challenged with *P. capsici*, **B** tomato challenged with *P. capsici*, and (**C**) potato challenged with *P. infestans*

Additionally, greenhouse pot experiments were conducted to investigate the disease control potential of SOJ5^V84F^ in pepper and tomato challenged with *P. capsici* and in potato challenged with *P. infestans.* Consistent with the results against *P. soaje*, pre-treatment with SOJ5 or SOJ5^V84F^ both significantly reduced the numbers of dead pepper (Fig. 5A), tomato (Fig. 5B), and potato plants (Fig. 5C) caused by *P. capsici and P. infestans*, and SOJ5^V84F^ exhibited a better protective effect than SOJ5 (Fig. 5A-C). Collectively, these results demonstrate that SOJ5^V84F^ acts as a dual-function biocontrol protein. It retains the ability to elicit immune responses in a wide range of plant hosts while also functioning as a dominant-negative inhibitor of sterol signaling in *Phytophthora*. This combination of immune stimulation and direct pathogen inhibition positions SOJ5^V84F^ as a promising candidate for protein-based disease control strategies.

## Discussion

Sterols are essential components of eukaryotic membranes and a well-established target for antifungal compounds. Most classic fungicides, such as azoles and polyenes, function by disrupting sterol biosynthesis or directly binding ergosterol in fungal membranes (Kazan and Gardiner 2017). For example, azoles are effective fungicides that target the sterol biosynthesis cytochrome P450s, but gene losses make sterol auxotrophic oomycetes escaping these fungicides (Judelson 2017). The antifungal herbicolin A produced by the biocontrol bacteria targets ergosterol to disrupt lipid raft (Xu et al. 2022). The amphotericin B and its derivate AM-2–19 extract ergosterol from lipid bilayers to kill fungi (Maji et al. 2023). Another strategy is targeting the sterol intracellular transport system. Sterol carrier protein-2 (SCP-2) and its homologs transfer sterols intracellularly and are widespread in eukaryotes (Galano et al. 2022). Several plant-derived natural products function as SCP-2 inhibitors and exhibit lethal effects in insects (Chaudhary and Gupta 2023). Likewise, oxathiapiprolin is an effective oomycete fungicide targeting the oxysterol binding protein (Pasteris et al. 2016). However, these fungicides are largely ineffective against oomycetes like *Phytophthora*, which are sterol auxotrophs and lack the ability to synthesize sterols de novo. Instead, these pathogens scavenge sterols from their environment or host during infection (Wang et al. 2021; Pei et al. 2024). This fundamental biological difference renders ergosterol-targeting fungicides useless against *Phytophthora* species and underscores the urgent need for novel strategies tailored to the unique sterol biology of oomycetes.

In this study, we exploited our previous discovery of the sterol-sensing pathway in *P. sojae*—mediated by the receptor-like kinase SSRK1 and elicitins—to design a targeted antimicrobial strategy (Pei et al. 2024). The engineered protein SOJ5^V84F^, bearing a point mutation in the sterol-binding pocket, retained its ability to interact with SSRK1 while losing sterol-binding activity. This feature allowed it to function as a dominant-negative competitor that blocked sterol-induced signaling and suppressed *Phytophthora* growth. Unlike chemical inhibitors that disrupt sterol biosynthesis, SOJ5^V84F^ interferes with the perception and utilization of host-derived sterols, representing a conceptual shift from targeting biosynthesis to targeting receptor-mediated sterol sensing.

Strikingly, SOJ5^V84F^ also retained its capacity to activate immune responses in multiple plant species, similar to canonical elicitins (Derevnina et al. 2016). This dual functionality—immune activation and pathogen inhibition—confers a unique advantage for disease control. The superior protective effect of SOJ5^V84F^ over wild-type SOJ5 in greenhouse assays suggests that blocking pathogen physiology while simultaneously priming host immunity may achieve synergistic protection. Interestingly, our strategy conceptually echoes the recently revealed dual-function mechanism of the host pathogenesis-related protein 1 (PR1). PR1 not only binds membrane sterols via its conserved CAP domain—thereby depriving sterol-auxotrophic pathogens like *Phytophthora* of essential membrane components—but also releases a C-terminal peptide (CAPE1) that acts as a damage-associated molecular pattern (DAMP) to activate immune responses (Chen et al. 2014; Breen et al. 2017; Gamir et al. 2017). Thus, PR1 exemplifies a natural dual-function defense protein, combining direct pathogen inhibition with host immune activation. In this context, SOJ5^V84F^ can be viewed as an engineered, pathogen-derived counterpart: it directly blocks sterol perception through dominant-negative competition for SSRK1 and concurrently activates conserved immune responses in plants. The convergence of function between a host-derived defense protein and a modified pathogen effector reinforces the potential of sterol-targeting and immune-eliciting proteins as a new class of biocontrol agents. From an application standpoint, recombinant SOJ5^V84F^ can be produced at scale in *E. coli* or yeast at relatively low cost, similar to current industrial enzyme production systems. The micromolar concentrations sufficient for pathogen inhibition and disease protection in our assays indicate that only modest application rates may be needed in the field. Furthermore, formulation strategies such as lyophilization or encapsulation can improve protein stability and facilitate large-scale deployment, highlighting the scalability of this biocontrol approach.

## Conclusion

Overall, our study builds upon the previously established sterol-sensing mechanism in *Phytophthora* and introduces a novel strategy to disrupt this pathway using a dominant-negative elicitin mutant. The engineered SOJ5^V84F^ protein effectively blocks pathogen sterol perception and suppresses growth, while simultaneously activating immune responses in host plants. Our findings demonstrate the feasibility of designing dual-function biocontrol proteins that directly target essential nutrient sensing pathways in pathogens while activating host defense. This work provides a conceptual and practical framework for protein-based disease control tailored to the unique biology of sterol-auxotrophic oomycetes.

## Materials and methods

### Expression and purification of recombinant proteins

Recombinant His-tagged SOJ5 and SOJ5^V84F^ proteins were expressed in *Escherichia coli* BL21 (DE3) cells. Protein expression was induced with 0.5 mM isopropyl β-D-1-thiogalactopyranoside (IPTG; Sangon Biotech, A600168) at 18 °C for 16 h. Bacterial cells were harvested and lysed by sonication in lysis buffer (20 mM Tris–HCl, pH 7.5, 0.5 M NaCl) at 4 °C. The recombinant proteins were purified using Ni–NTA Sepharose affinity chromatography as previously described (Pei et al. 2024).

### Antimicrobial assays

The *P. sojae* wild-type strain P6497 and *ssrk1*^△^ mutant were maintained on V8 agar medium at 25 °C in the dark. Prior to antimicrobial assays, all strains were subcultured twice on sterol-free minimal medium to deplete endogenous sterol reserves from the mycelia. Antimicrobial assays were performed in 10 mL Erlenmeyer flasks containing minimal medium supplemented with 5 μM *β*-sitosterol and 10 mM Tris–Cl (pH 7.5). Six actively growing mycelial plugs were inoculated into each flask and incubated at 25 °C on a rotary shaker at 75 rpm for 10 days. Fungal biomass was then determined by measuring the dry weight. Mycelia were harvested by filtration, washed with distilled water, dried at 60 °C to constant weight, and weighed using an analytical balance.

### Calcium influx assays

Protoplasts of *P. sojae* were pre-treated with or without SOJ5^V84F^ protein (4 μM) for 30 min, followed by the addition of *β*-sitosterol (25 μM) or ethanol (mock) for another 30 min. Subsequently, protoplasts were incubated with 2 μM Fluo-4 AM (Beyotime, S1060) in the dark at 25 °C for 30 min. For real-time calcium influx measurements, the protoplasts were transferred into calcium-free minimal medium, and fluorescence signals were recorded using a microplate reader (Spectra Max M5, Molecular Devices, USA) at 494 nm excitation and 516 nm emission over a 900 s period.At 60 s, CaCl₂ (2 mM) was added along with ethanol (mock), *β*-sitosterol (25 μM), or *β*-sitosterol combined with SOJ5^V84F^. Changes in intracellular calcium levels were indicated by variations in Fluo-4 AM fluorescence intensity. Fluorescence values were normalized to those of the mock control and presented as relative fluorescence units (RFU). All assays were performed in triplicate and independently repeated at least three times.

### MAPK phosphorylation

Seven-day-old *P. sojae* mycelia grown in liquid minimal medium were collected and pre-treated with or without SOJ5^V84F^ protein (4 μM) for 30 min, followed by exposure to *β*-sitosterol (25 μM) for 15 min. Mycelia treated with ethanol served as mock controls. After treatment, total proteins were extracted using homogenization buffer containing 50 mM HEPES (pH 7.5), 250 mM sucrose, 15 mM EDTA, 5% (w/v) glycerol, 0.5% (w/v) polyvinylpyrrolidone, 3 mM DTT, and 1 × protease inhibitor cocktail. Protein samples were separated by 12% SDS-PAGE and transferred to PVDF membranes. Membranes were blocked with TBS containing 0.01% Tween-20 and probed with a rabbit monoclonal anti-phospho-p44/p42 MAPK antibody (1:5,000 dilution; Cell Signaling Technology). After washing, membranes were incubated with HRP-conjugated goat anti-rabbit IgG secondary antibody (Jackson ImmunoResearch), and signals were detected using Pierce ECL Substrate (Thermo Scientific)..

### Measurement of reactive oxygen species

Reactive oxygen species production was monitored with an L-012/peroxidase-based assay on leaf discs collected from soybean, pepper, tomato, and potato according to previously described methods (Pei et al. 2023). The leaf discs were floated overnight on 200 μL of ddH_2_O in a 96-well plate. The ddH_2_O was replaced with a working solution [20 μM L-012 (Waco), 20 μg/mL peroxidase (Sigma-Aldrich), and 4 μM purified SOJ5 or SOJ5^V84F^ protein. After the addition of the working solution, the plate was immediately moved to a GLOMAX96 microplate luminometer (Promega, Madison, WI, USA) for measurement of luminescence.

### Virulence assays

Zoospores of *P. sojae* were induced according to previously described methods (Pei et al. 2022) and diluted to a concentration of 200 zoospores/10 μL. Etiolated seedlings were inoculated by pipetting 10 μL of the zoospore suspension onto the soybean hypocotyls, followed by growth at 25 °C in a dark incubator with 80% relative humidity. Lesions were assessed and photographed at 2 days post-inoculation (dpi). Virulence was also quantified by determining the ratio of *P. sojae* DNA to soybean DNA in infected soybean samples using qPCR analysis.

For soybean pot assays, soybean plants (Hefeng 47) were grown in pots in a glasshouse. SOJ5 or SOJ5^V84F^ proteins (1 μM) were sprayed onto seedling hypocotyls 12 h before inoculation with a piece of mycelium in agar, followed by incubation in a growth chamber at 23 °C for 48 h, after which disease ratings were made. For assaying protection from *P. capsici* infection, 4 μM SOJ5 or SOJ5^V84F^ protein was sprayed to the point of run-off on pepper or tomato seedlings 12 h prior to inoculation. Then the seedlings were inoculated with a spore suspension of *P. capsici* and incubated in a growth chamber at 23 °C for 5 days, after which disease ratings were made. Similarly, for assaying protection of potato from *P. infestans*, 2 μM SOJ5 or SOJ5^V84F^ protein was sprayed to the point of run-off on potato seedlings 12 h prior to inoculation. Then the seedlings were inoculated with a sporangial suspension of *P. infestans* and incubated in a growth chamber at 20 °C for 5 days, after which disease ratings were made.

### RNA isolation and quantitative reverse transcription PCR

Total RNA was extracted from all samples using the PureLink™ RNA Mini Kit (Thermo Fisher Scientific), following the manufacturer’s instructions. Approximately 900 ng of total RNA was used for first-strand cDNA synthesis with oligo(dT) primers. The resulting cDNA was diluted threefold, and 2 μL of the diluted cDNA was used as the template in a 20 μL quantitative PCR reaction containing SYBR Green Master Mix (Vazyme). Quantitative real-time PCR (qRT-PCR) was performed using the ABI Prism 7500 Fast Real-Time PCR System (Applied Biosystems) according to the manufacturer's protocol. Gene expression levels were calculated using the comparative Ct method (2^−ΔΔCt^). All reactions were conducted in technical triplicates unless otherwise specified.

## Data Availability

The data generated or analyzed in this study are in the published article. The data that support the findings of this study are available from the corresponding author upon reasonable request.

## Abbreviations

*SSRK1*: Sterol-sensing receptor-like kinase 1
*MAMP*: Microbe-associated molecular pattern
*DAMP*: Damage-associated molecular pattern
*ELR*: Elicitin response
*REL*: Responsive to elicitins
*WT*: Wild-type
*ROS*: Reactive oxygen species
*SCP-2*: Sterol carrier protein-2
*PR1*: Pathogenesis-related protein 1
*CAPE1*: C-terminal peptide
*RFU*: Relative fluorescence units
*dpi*: Days post-inoculation
*qRT-PCR*: Quantitative real-time PCR
